# Single-Cell RNA-Sequencing Analyses Revealed Heterogeneity and Dynamic Changes of Metabolic Pathways in Astrocytes at the Acute Phase of Ischemic Stroke

**DOI:** 10.1155/2022/1817721

**Published:** 2022-04-30

**Authors:** Hongyu Ma, Yu Zhou, Zifu Li, Luojiang Zhu, He Li, Guanghao Zhang, Jing Wang, Haiyi Gong, Da Xu, Weilong Hua, Pei Liu, Xiaoxi Zhang, Yongxin Zhang, Lei Zhang, Bo Hong, Wang Zhou, Pengfei Yang, Jianmin Liu

**Affiliations:** ^1^Neurovascular Center, Changhai Hospital, Naval Medical University, Shanghai, China; ^2^Department of Orthopaedic Oncology, Shanghai Changzheng Hospital, Naval Medical University, Shanghai, China; ^3^Department of Urology, Eastern Hepatobiliary Surgery Hospital, Naval Medical University, Shanghai, China

## Abstract

Astrocyte plays important roles in the pathogenesis of ischemic stroke and reperfusion injury. They intensively participate in the energy metabolism of the brain, while their heterogeneity and function after ischemic stroke remain controversial. By employing single-cell sequencing of mice cortex at 12 h after transient middle cerebral artery occlusion (tMCAO) and comparing with the similar published datasets of 24h after tMCAO, we uncover the cellular phenotypes and dynamic change of astrocytes at the acute phase of ischemic stroke. In this study, we separately identified 3 major subtypes of astrocytes at the 12 h-tMCAO-system and 24 h-tMCAO-system, indicated the significant differences in the expression of genes and metabolic pathways in the astrocytes between the two time nodes after ischemic stroke, and detected the major change in the energy metabolism. These results provided a comprehensive understanding of the characteristic changes of astrocytes after ischemic stroke and explored the potential astrocytic targets for neuroprotection.

## 1. Introduction

Ischemic stroke is one of the leading causes of death globally [1, 2]. In recent years, multiple clinical trials had demonstrated the significant efficacy of endovascular therapy in large-vessel ischemic stroke; however, the percentage of patients with a good prognosis was still low after successful artery recanalization [3–8]. The blood reflow alone is unable to salvage all the damage in ischemic penumbra or volume of hypoperfused. In these tissues, a shortage of glucose and oxygen supply leads to the disruption of energy metabolism [9]. Further studies to explore the underlying mechanism of the energy metabolism dysfunction after ischemic stroke and reperfusion injury (IRI) are potential.

Astrocyte, as the most abundant subtype of brain cells, is critical for the regulation of brain energy metabolism [10]. Glutamate uptake can trigger astrocytic aerobic glycolysis and lead to the release of lactate. The lactate can be transported to neurons for oxidative phosphorylation, which is regarded as a vital energy source for active neurons [13–15]. The dynamic change of metabolism dysfunction in astrocytes induced by ischemic stroke was unclear. To reveal the heterogeneity and specific gene expressions of astrocyte activated by ischemic stroke is important to unveil this metabolism dysfunction procedure. The present categorizing of reactive astrocytes in pathological conditions was controversial, and the understanding of astrocytic heterogeneity in response to ischemic stroke was limited [16, 17].

In recent years, single-cell RNA-sequencing (scRNA-seq) provided new insight into the heterogeneities of subpopulations among various tissues. To uncover the astrocyte diversity and function states at the acute phase after ischemic stroke, together with a comprehensive analysis of the metabolism change after reperfusion, we performed the scRNA-seq using the brain cortex isolated from mice who got reperfusion 12 h after transient middle cerebral artery occlusion (tMCAO). These data were compared with the scRNA-seq data of 24 h after tMCAO obtained from a previous study, to further analyze the change of gene expression at 12 h and 24 h after reperfusion [18].

Our results revealed the relative heterogeneities of astrocyte subpopulations. We mainly found that the temporal dynamic of reactive astrocytes contributes to the pathogenesis of ischemic stroke at the acute phase. We illustrated the energy mentalism states of astrocytes at 12 h and 24 h after ischemic stroke and reperfusion and also identified the major altered genes and activated pathways of the reactive astrocytes at the two different time nodes. This study also could provide novel therapeutic targets for neuroprotection.

## 2. Materials and Methods

### 2.1. Animals

C57BL/6 N male mice with 8-week age and 20-24 g weigh were derived from the Beijing Vital River Company. All mice were reared in a pathogen-free SPF animal room at 20-26°C and 40-60% humidity under a 12-h light/dark cycle. Animals were randomly assigned into two groups and treated with either transient middle cerebral artery occlusion model (tMCAO) or sham surgeries. This study was approved by the ethics committee of the First Affiliated Hospital of Naval Medical University. All the samples acquisition and experimental procedures were performed in compliance with the animal care standards of the National Institutes of Health Guide.

### 2.2. Transient Middle Cerebral Artery Occlusion Model

Anesthesia was induced by inhalation of 5% isoflurane and maintained by inhalation of 2.0% isoflurane with a face mask. The mice were placed on the heat mat in a supine position, and the body temperature was maintained at 36.5 ± 0.5°C, which was verified using a rectal probe. After shaving the mouse's neck and disinfection with 70% ethyl alcohol, we made a para-midline incision and exposed the left common carotid artery (CCA), internal carotid artery (ICA), and external carotid artery (ECA). Then we make a small incision in the ECA and insert a suture embolus. The suture embolus was guided through the ICA until slight resistance was felt in the left middle cerebral artery. Dynamic change of cerebral blood flow (CBF) in the operational brain region was monitored by the laser speckle flowmetry. The focal cerebral ischemia (CBF <30%) was produced for 120 min, and the suture embolus was withdrawn to restore CBF. For the sham group, mice were treated with surgical procedures under the same anesthesia, in which the MCAO procedure was waived.

### 2.3. Brain Tissue Dissociation for 10x Genomics

The mice were killed under anesthesia 12 hours after CBF reperfusion. The whole brain was resected, and the cortex of the left brain was separated and minced using a Vannas scissor. Then the cortex tissue was digested with papain and incubated for 10 minutes at a 37°C constant temperature bath and was gently mixed using a pipette after each incubation. After that, the tissue fragments were filtered with a 70-m strainer and resuspended with ice-cold Dulbecco phosphate-buffered saline (D-PBS). The cell suspension was centrifuged at 300 *g* for 10 min at 4°C. The supernatant was abandoned, and the myelin sheath debris in the remaining tissue was removed with 900 *μ*L debris removal solution plus 3.1 mL D-PBS. After centrifugation at 3000 *g* for 10 min, the cell pellet in the lower layer was obtained. The blood cells in the cell pellet were removed using cold blood cell removal solution. Then the cells were resuspended with D-PBS plus 0.04% bovine serum albumin (BSA) for further cell counting. The trypan blue staining was performed to evaluate the number and viability of the cells. More than 100000 cells were obtained from each brain sample, and the average cell viability in the sham group and MCAO group were >85% and >50%, respectively.

### 2.4. Single-Cell RNA-Sequencing Using 10x Genomics Platform

The construction of the Single Cell Library was performed under the manufacturer's instruction of Chromium Next GEM Single Cell V(D)J Reagent Kits v1.1. Briefly, the single-cell gel beads-in-emulsion (GEM) was generated by combining the Master Mix with cells, Gel Beads, and Partitioning Oil using Chromium Next GEM Chip G. The 10× Barcoded, full-length cDNA was acquired from the incubation of GEM. After the cleanup of the first-strand cDNA with Silane magnetic beads, the cDNA was amplificated by PCR to generate sufficient material for library construction. The P5 primer, P7 primer, and Illumina R2 sequence (read 2 primer sequence) were used to construct the final library, and the libraries were sequenced on the NovaSeq 6000 system. The 10x Genomics Cell Ranger (v3.0.2) pipeline (10x Genomics, Pleasanton, California) was used to generate de-multiplex samples, process barcodes, and align and filter reads and the output of feature barcode matrices. All these procedures were performed under the manufacturer's instructions.

### 2.5. The Data Source of scRNA-Seq Database and Characteristics of Samples

The RNA-sequencing data enrolled in this analysis were consisted of two parts. The first part was the results of the above single-cell RNA-sequencing, which were derived from 3 control brain cortex samples and 3 samples treated with 12 h-tMCAO (reperfusion for 12 h after the 2 h transient occlusion of the middle cerebral artery). The second part was downloaded from the GEO database (10x Genomics data, GEO: GSE174574). These data were obtained from 3 control brain cortex samples and 3 samples treated with 24 h-tMCAO (reperfusion for 24 h after the 1 h transient occlusion of the middle cerebral artery). Based on these two parts of data, a total database and two data systems, 12 h-tMCAO-system and 24 h-tMCAO-system, were constructed. The characteristic of samples used in the two systems was close. In the 12 h-tMCAO-system, the brain cortex samples were dissected from 8-week-old (56-65 days) C57BL/6 N male mice weighing 20-24 g, while the samples in the 24 h-tMCAO-system were from C57BL/6 J male mice aging 60-70 days and weighing 20-25 g. The model-making methods and sampling sites were similar. The major differences between the 12 h-tMCAO-system and the 24 h-tMCAO-system were the periods of ischemia (2 h versus 1 h) and reperfusion (12 h versus 24 h). The 12 h-tMCAO-system, with longer ischemia time and less reperfusion time, probably represented a worse injury state of brain tissue, while 12 h-tMCAO-system may represent a recovery state. The data in the two systems were analyzed independently, while the method for the analyses were the same.

### 2.6. Quality Control and Dimensionality Reduction

The original data were imported into the Seurat (v3) R (4.0.3) toolkit. All these data underwent the same standard for quality control and normalization. Low-quality cells (<200 genes/cell and >10% mitochondrial genes) and doublets (assuming 7.5% doublet formation rate) were removed. For the total database, the datasets from 12 samples were integrated using the Harmony algorithm to remove the batch effect. For the respective analysis of 12 h-tMCAO-system and 24 h-tMCAO-system, the data from our 6 samples and the data from 6 published samples were separately integrated.

### 2.7. Clustering and Cell Subtypes Identification

The feature selection and identification of the highly variable genes were conducted using the Seurat function “FindVariableFeatures.” The top 2000 genes with the highest cell-to-cell variation were used for data integration. The “ScaleData” function and “RunPCA” function were used for data scaled and principal component analysis (PCA). The auto-clustering analyses were performed using the “FindNeighbors” and “FindClusters” functions. The UMAP scatter plot was used for the visualization of the clustering results. The major cell subtypes were identified using known marker genes. The significantly highly expressed marker genes in each identified cluster were also identified for verification of the cell types annotation. The top 5 most significant genes and the proportion of each sample in identified clusters were displayed with a heatmap.

### 2.8. Gene Expression and Metabolic Pathways Analyses

To further evaluate the heterogeneity of astrocytes in ischemic stroke, the subclustering of astrocytes was performed using the monocle3 package in R, and the differentiation of subpopulations was analyzed with the pseudo-time track. The average expression count for each gene per cluster was calculated using gene set variation analysis (GSVA) method. The transcription factor and cell states were analyzed with single-cell regulatory network inference and clustering (SCENIC) method [19]. To further analyze the change of energy metabolism between 12 h and 24 h after reperfusion of stroke, the expression of genes for key enzymes or transporters in glycolysis, aerobic oxidation, glycogen metabolism, energy metabolites transportation, and fatty acid metabolism were analyzed. The critical genes for each procedure were identified and listed in Table 1.

### 2.9. Immunofluorescence

Immunofluorescence staining was performed to verify the protein level expression of genes for energy metabolism in astrocytes. The paraffin sections of the brain cortexes from three cases (control, 12 h reperfusion after ischemia, and 24 h reperfusion after ischemia) were deparaffinized and rehydrated using xylene and gradient ethanol and washed with distilled water. Then they were immersed in EDTA antigen retrieval buffer (pH 8.0) and were set in a sub-boiling temperature for 8 min, standing for 8 min, and then followed by another sub-boiling temperature for 7 min for antigen retrieval. Next, the objective tissue was marked with a liquid blocker pen, and 3% BSA was added to cover the marked tissue to block nonspecific binding for 30 min. Then the sections were incubated with primary antibodies (diluted with PBS appropriately) overnight at 4°C. The following primary antibodies were used: mouse anti-GFAP (No. ab4648, monoclonal, Abcam, 1 : 500), rabbit anti-GLUT1 (No. ab115730, monoclonal, Abcam, 1 : 400), rabbit anti-Lactate Dehydrogenase (No. ab52488, monoclonal, Abcam, 1 : 100), rabbit anti-PKM (No. ab150377, monoclonal, Abcam, 1 : 100), and rabbit anti-EAAT1 (No. ab176557, monoclonal, Abcam, 1 : 100). Then the sections were washed with PBS (pH 7.4) and incubated with secondary antibody: goat anti-mouse 488-conjugated goat anti-mouse IgG (H + L) (GB25301, Servicebio, 1 : 400) and Cy3 conjugated goat anti-rabbit IgG (H + L) (GB21303, Servicebio, 1 : 300). Then the sections were incubated with DAPI solution at room temperature for 10 min in a dark place for DAPI counterstain in the nucleus. The images were detected and collected by fluorescent microscopy, where DAPI glows blue, fluorescein isothiocyanate (FITC) glows green, and Cy3 glows red.

## 3. Results

### 3.1. Single-Cell RNA-Sequencing from Sham and tMCAO Cortices Identifies Major Cell Types

**Figure 1:**
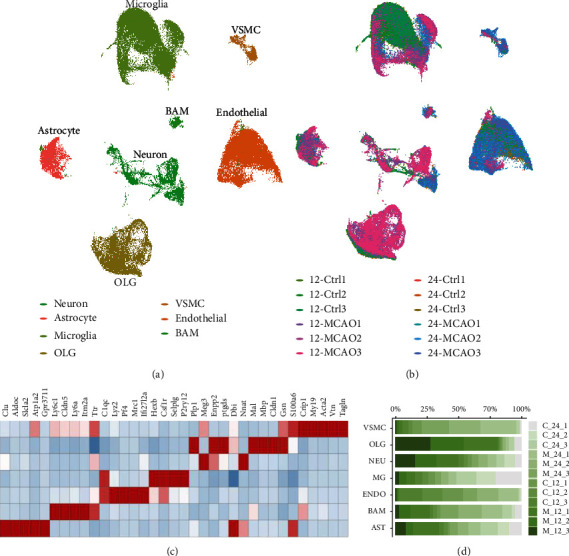
Major brain cell subtypes and genes signature. (a). UMAP plot of 104382 cells to visualize subtypes of brain cells based on the expression of known marker genes. (b). The UMAP plot visualized by sample distribution. (c). Heatmap of the relative expression level of genes across cell types. (d). The proportion of cells that contributed to each cluster by each sample, colored by cell type.

Twelve brains cortexes samples (6 of control and 6 of tMCAO) undergoing scRNA-sequencing 10X Genomics platforms were included. After quality control, cells from 12 samples were integrated using the harmony algorithm, in which 104382 cells (49588 from control and 54794 from tMCAO) were selected for unsupervised clustering and t-distributed stochastic neighbor embedding (tSNE). Brain cell clusters were identified using well-known marker genes (Figures 1(a) and 1(b)). The major cell subtypes included astrocyte (characterized by *Gfap*, *Aqp4*, *Mfge8*, and *Aldh1l1*), microglia (characterized by *P2ry12*, *Tmem119*, *Tnf*, and *Gpr84*), border-associated macrophage (BAM, characterized by *Lyz2*, *Mrc1*, *Cd38*, and *H2-Aa*), oligodendrocyte (OLG, characterized by *Fa2h* and *Olig1*; OPC, oligodendrocyte precursor cell, characterized by *Pdgfra* and *Cspg4*), endothelial cells (characterized by *Cldn5*, *Flt1*, *Esam*, and *Ly6c1*), and vascular smooth muscle cells (VSMCs, characterized by *Rgs5*, *Vtn*, *Myl9*, and *Col4a1*)). Scaled normalized gene expression in the major cell subtypes was displayed in a heatmap. For each cell cluster identified, the proportion of cells in each sample was shown in Figure 1(c).

**Figure 2:**
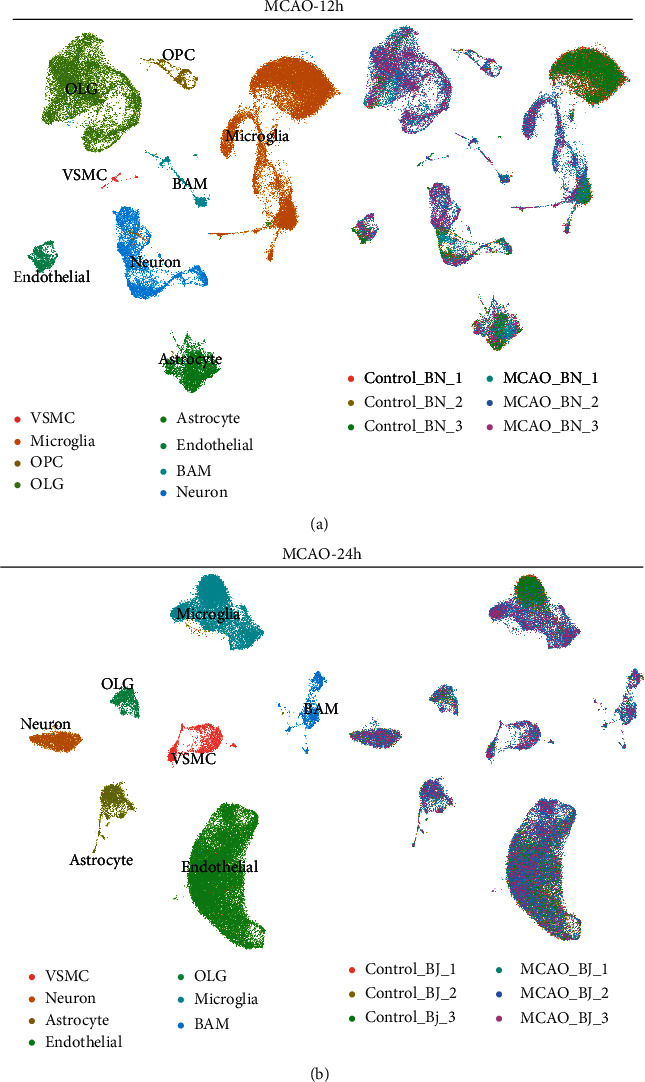
Major brain cell subtypes in the 12 h-tMCAO-system and 24 h-tMCAO-system. (a). UMAP plot to visualize subtypes of brain cells based on the expression of known marker genes (left) and the sample distribution (right) in 12 h-tMCAO-system. (b). UMAP plot to visualize subtypes of brain cells based on the expression of known marker genes (left) and the sample distribution (right) in 24 h-tMCAO-system.

To further analyze the cell heterogeneity and function change between 12 h and 24 h after tMCAO, the following analyses were performed based on the 12 h-tMCAO-system and 24 h-tMCAO-system. These samples were separately integrated, reclustered, and analyzed using the same method (Figure 2). The total cells in the 12 h-tMCAO-system were 54775 and in the 24 h-tMCAO-system were 49607. The proportions of astrocytes in the two systems were comparable (8% versus 6%). In the 12 h-tMCAO-system, microglia (46% versus 24%) and OLG (24% versus 3%) accounted for higher proportions compared with 24 h-tMCAO-system, while the endothelial cells (3% versus 51%) were lower.

### 3.2. Astrocyte Heterogeneity in the 12h-tMCAO-System

**Figure 3:**
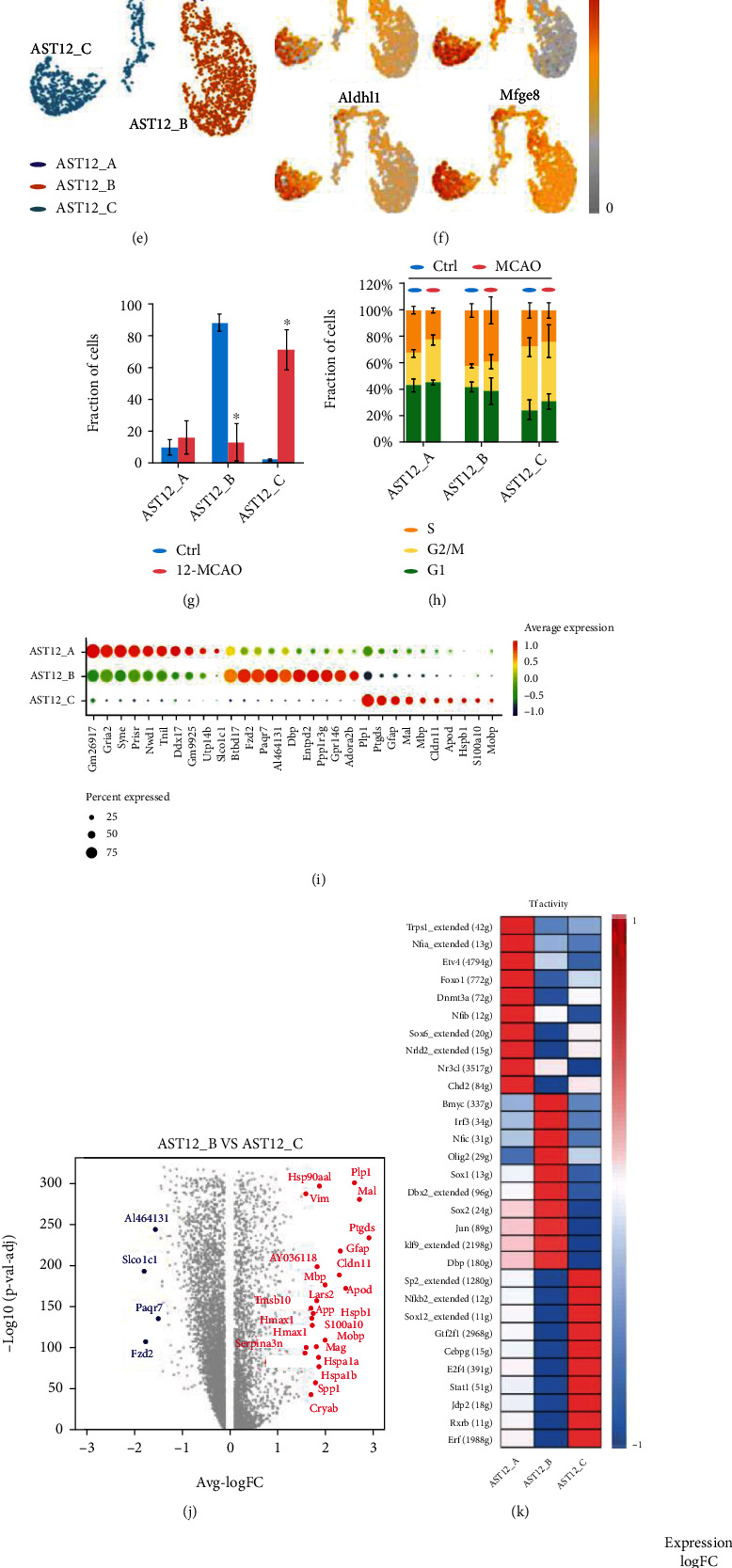
Astrocyte heterogeneities in the 12 h-tMCAO-system. (a). Unsupervised clustering of astrocytes visualized by sample distribution. (b). Visualization of the expression of signature genes of astrocytes. (c). Subpopulation of astrocytes using monocle algorithm visualized by sample distribution. (d). The t-SNE plot for cell differentiation trajectory. (e). Identified subpopulation of astrocytes clustered by monocle algorithm. (f). Visualization of the expression of previously reported signature genes of astrocytes in the t-SNE plot clustered by monocle algorithm. (g). The proportion of cells that contributed to each cluster by control and MCAO. (h). The cell cycle distribution in the three subpopulations. (i). Bubble plots displayed the top 10 signature genes for each subpopulation. (j). Volcano plot to identify the differential genes between AST12_B and AST12_C. (k). Heatmap of the expression regulation by transcription factors of the identified clusters. (l). Gene ontology network based on genes that are highly upregulated.

To identify the heterogeneity of astrocytes at 12 h after ischemic stroke, the clusters identified as astrocytes in the 12 h-tMCAO-system were further analyzed. Firstly, we conducted unsupervised clustering of astrocytes pooled from the total cells, but no new subset was generated. (Figure 3(a)). The expression of previously reported signature genes of astrocytes was examined and displayed (Figure 3(b)). Then subclustering of astrocytes was performed using the monocle3 package in R, revealing 3 subset clusters named AST12_A, AST12_B, and AST12_C. (Figures 3(c) and 3(e)). Most of these cells were classified into AST12_B and AST12_C. The proportion of cells from control samples was much higher in AST12_B than in AST12_C, and the situation inversed for MCAO samples (Figure 3(g)). The cell cycle distribution in the three subpopulations was similar (Figure 3(h)). As we can see, the astrocytes experienced the process from a static state (AST12_A) to two functional states (AST12_B and AST12_C), indicated by the continuous change of differentiation in pseudo-time trajectory and the expression of reactive markers genes (Figures 3(d) and (f)). Bubble plots displayed the top 10 signature genes for each subpopulation (Figure 3(i)). The highly expressed genes in AST12_B were *Slco1c1*, *Btbd17*, *Fzd2*, *Paqr7*, and *Al464131*, while in AST12_C were *Plp1*, *Ptgds*, *Gfap*, *Mal*, and *Mbp*. Differentially expressed genes between AST12_B and AST12_C were displayed with a volcano plot (Figure 3(j)). There were numerous genes significantly increased in AST12_C including *Vim*, *Hsp90aa1*, *Plp1*, *Mal*, and *Ptgd*, whereas *Slco1c1*, *Paqr7*, *Al464131*, and *Fzd2* were significantly downregulated. The transcription factors were analyzed with the single-cell regulatory network inference and clustering (SCENIC) method. Transcription factors including Trps1, Nfia, and Etv4 were upregulated in AST12_A, and Bmyc, Irf3, and Nfic were greatly increased in AST24_B, and Sp2, Nfkb2, and Sox12 were highly upregulated in AST24_C (Figure 3(k)). Specific-gene ontology network revealed major pathways activated at the 12 h-tMCAO-system including oxidative phosphorylation, ferroptosis, HIF-1 pathway, chemokine signaling pathway, AGE-RAGE signaling pathway, and protein processing in endoplasmic reticulum. (Figure 3(l)).

### 3.3. Astrocyte Heterogeneity in the 24h-tMCAO-System

**Figure 4:**
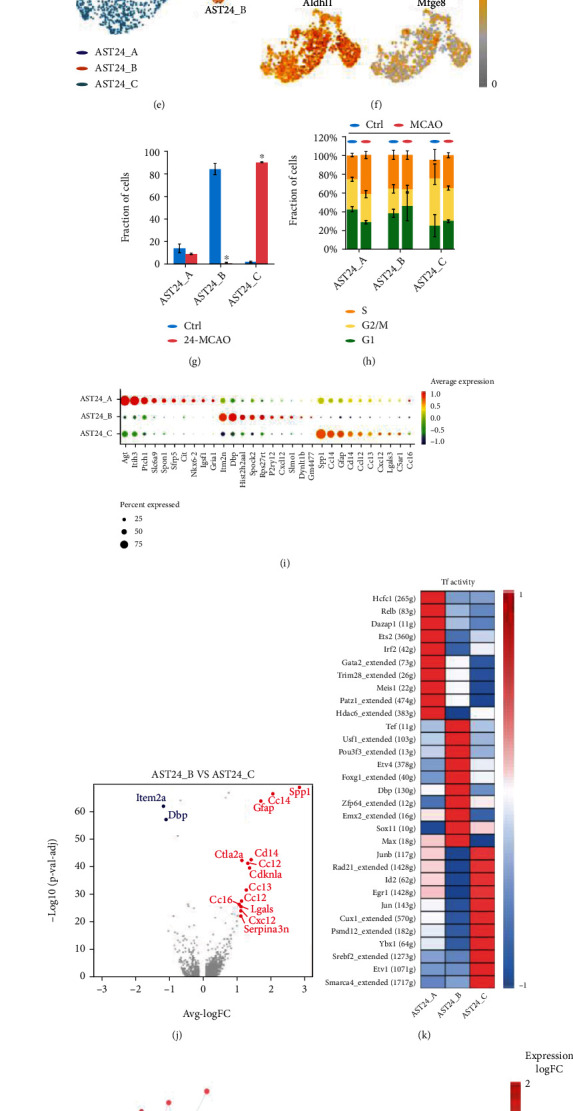
Astrocyte heterogeneities in the 24 h-tMCAO-system. (a). Unsupervised clustering of astrocytes visualized by sample distribution. (b). Visualization of the expression of signature genes of astrocytes. (c). Subpopulation of astrocytes using monocle algorithm visualized by sample distribution. (d). The t-SNE plot for cell differentiation trajectory. (e). Identified subpopulation of astrocytes clustered by monocle algorithm. (f). Visualization of the expression of previously reported signature genes of astrocytes in the t-SNE plot clustered by monocle algorithm. (g). The proportion of cells that contributed to each cluster by control and MCAO. (h). The cell cycle distribution in the three subpopulations. (i). Bubble plots displayed the top 10 signature genes for each subpopulation. (j). Volcano plot to identify the differential genes between AST24_B and AST24_C. (k). Heatmap of the expression regulation by transcription factors of the identified clusters. (l). Gene ontology network based on genes that are highly upregulated.

The subclusters of astrocytes in the 24 h-tMCAO-system were similar to that in the 12 h-tMCAO-system. There was no new subset generated either after unsupervised clustering of astrocytes. (Figure 4(a)) The distribution of signature genes of astrocytes was displayed. (Figure 4(b)) AST24_A, AST24_B, and AST24_C were identified after clustering using the monocle3 package in R. (Figures 4(c) and 4(e)). The fraction of the three subpopulations was similar to that in the 12 h-tMCAO-system. (Figure 4(g)) The cell cycle distribution in the three subpopulations was also similar (Figure 4(h)) The pseudo-time trajectory and signature genes distribution also suggested that the astrocytes in the 24 h-tMCAO-system were differentiated from a static state (AST24_A) into two different functional states (AST24_B andAST24_C). (Figures 4(d) and 4(f)). Bubble plots suggested that the signature genes of subpopulations in the 24 h-tMCAO-system were not similar with those in the 12 h-tMCAO-system, in which *Itm2a*, *Dbp*, *Hist2h2aa1*, *Spock2*, and *Rps27rt* were highly expressed in AST24_B, while *Spp1*, *Ccl4*, *Gfap*, *Cd14*, and *Ccl12* were highly expressed in AST24_C (Figure 4(i)). The common genes highly expressed in both two systems were *Dbp* (for subpopulation B) and *Gfap* (for subpopulation C). This discrepancy could have been due to the different pathological states between 12 h and 24 h after tMCAO. The volcano plot showed the significantly increased expression of *Spp1*, *Ccl2*, *Ccl4*, *Gfap*, and *Cd14* in AST24_C and the significant downregulation of *Item2a* and *Dbp* when compared with AST24_B. There were more upregulated genes related to cells proliferation such as *Vim*, *Plp1*, and *Mal* in AST12_C compared with AST12_B, while in the 24 h-tMCAO-system AST24_C highly expressed genes related to cytokines, such as *Ccl2*, *Ccl3*, *Ccl4*, and *Cxcl2*. The heatmap of SCENIC suggested that transcription factors such as Hcfc1, Relb, and Dazap1 were highly upregulated in AST24_A, while Tef, Usf1, and Pou3f3 were greatly increased in AST24_B, and Junb, Rad21, and Id2 were upregulated in AST24_C. (Figure 4(j)). Gene ontology network suggested that more inflammation pathways were activated at 24 h-tMCAO-system, such as cytokine-cytokine receptor interaction, chemokine signaling pathway, TNF signaling pathway, NF-*κ*B, and IL-17 signaling pathway. (Figure 4(k)). We also detected the activation of the HIF-1 pathway and AGE-RAGE signaling pathway, which were also activated at the 12-tMCAO-system. (Figure 4(l)).

### 3.4. Expression Heterogeneity and Relationship between 12h and 24h after tMCAO

**Figure 5:**
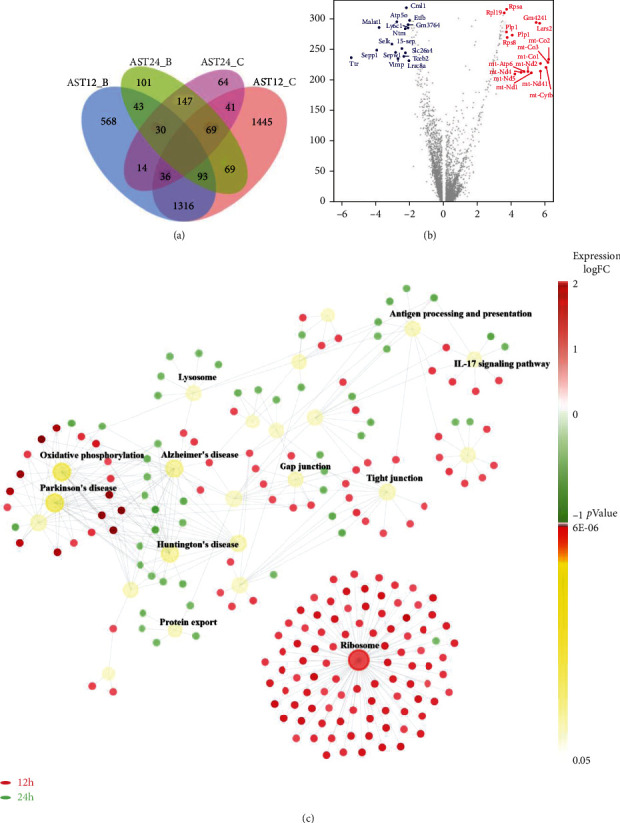
Differential expression and relationship between 12 h and 24 h after tMCAO. (a). Genes that were significantly upregulated in four astrocytic subsets are compared using Venn diagram. (b). Volcano plot displayed the differential genes of reactive astrocytes between 12 h and 24 h. (c). Astrocytic gene ontology network showed the highly activated pathways at 12 h and 24 h after MCAO.

To assess the time-related heterogeneity of astrocytes, we compared the expression of signature genes in four functional astrocytes types. The total differential genes were displayed in the Venn diagram. AST12_C showed the highest number of uniquely upregulated genes (*n* = 1445), whereas the number in AST24_C was the lowest (*n* = 64). (Figure 5(a)). The differential genes of reactive astrocytes between 12 h and 24 h were further displayed using a volcano plot. Compared with the astrocytes at 24 h, more mitochondria genes were upregulated at 12 h, which may suggest that the proportion of dying cells was higher or the energy metabolism dysfunction was severer at 12 h (Figure 5(b)). Astrocytic gene ontology network showed that both the pathways related to gap junction and tight junction were activated at 12 h, which may imply the activation of blood-brain barrier and intercellular connection, while these activations may decrease at 24 h. The oxidative phosphorylation pathway upregulated at 12 h, suggesting the energy deficiency and the increasing energy demand in astrocytes, while protein export, lysosome, and antigen processing and presentation pathways were upregulated at 24 h, probably indicating the enhancing interaction between astrocytes and microenvironment. (Figure 5(c)).

### 3.5. Energy Metabolism Dynamics in Astrocytes after Ischemic Stroke

**Figure 6:**
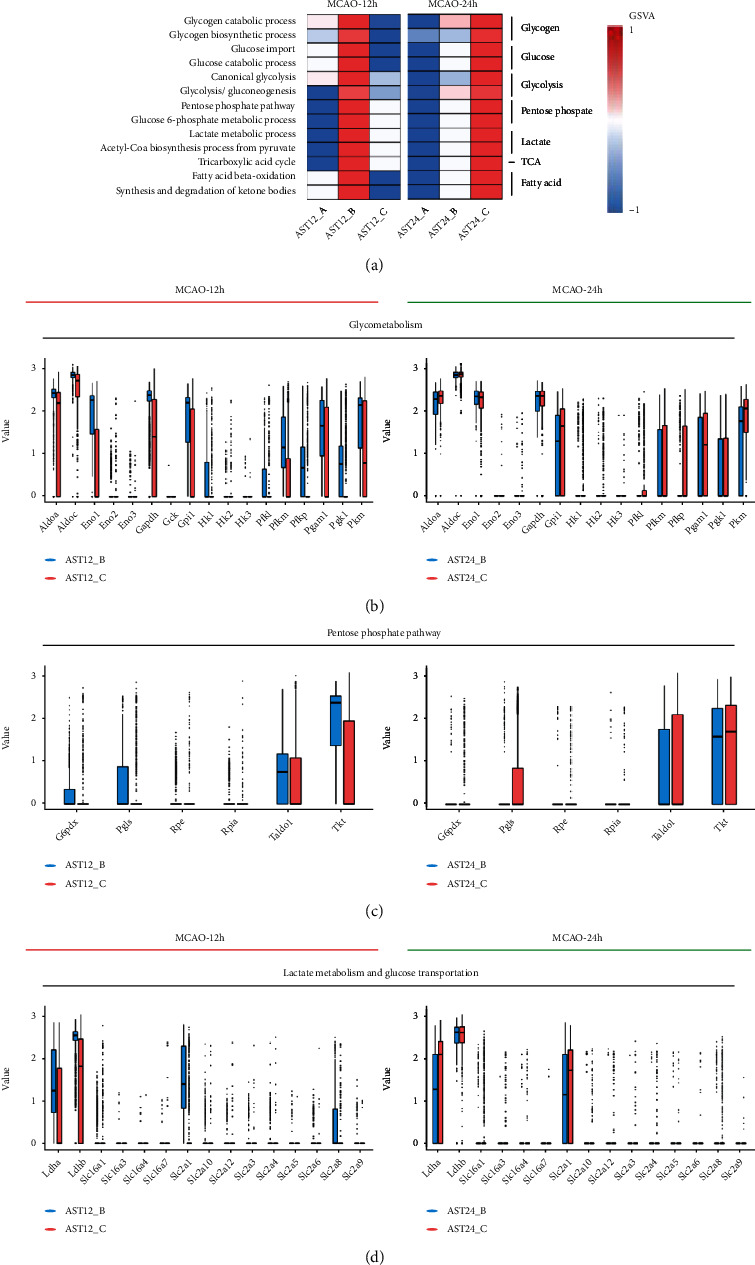
Comparison of the major gene expression in energy metabolic pathways in astrocytes at 12 h and 24 h after MCAO. (a). Heatmap of GSVA results indicated major changes in the energy metabolism pathways. (b). Boxplots indicated the changes of expression of genes related to glycolysis. (c). Boxplots indicated the changes of expression of pentose phosphate pathway. (d). Boxplots indicated the changes of expression of the syntheses and transportation of lactate and glucose transporters.

**Figure 7:**
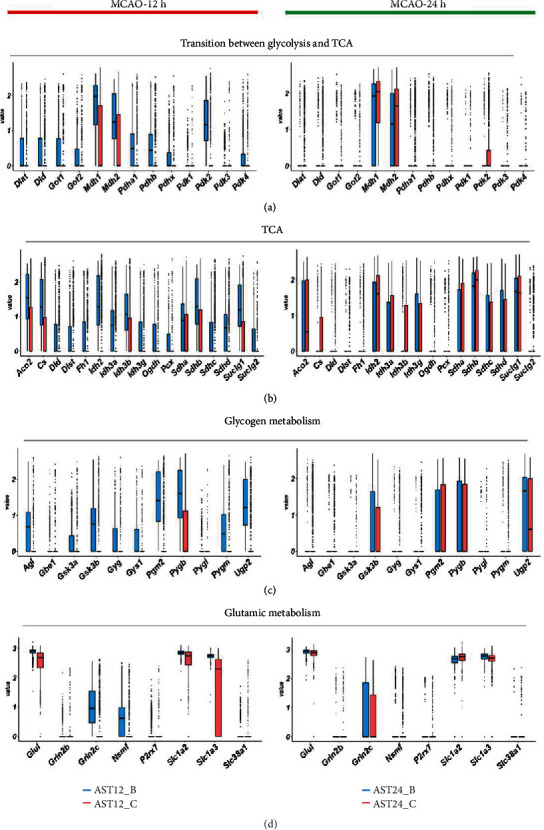
Comparison of the gene expression in energy metabolism. (a). Boxplots indicated the change of expression of genes for the transition between glycolysis and tricarboxylic acid cycle (TCA). (b). Boxplots indicated the change of expression of genes for TCA. (c). Boxplots displayed the change of expression of genes for glycogen metabolism. (d). Boxplots displayed the change of expression of genes for glutamic metabolism.

**Figure 8:**
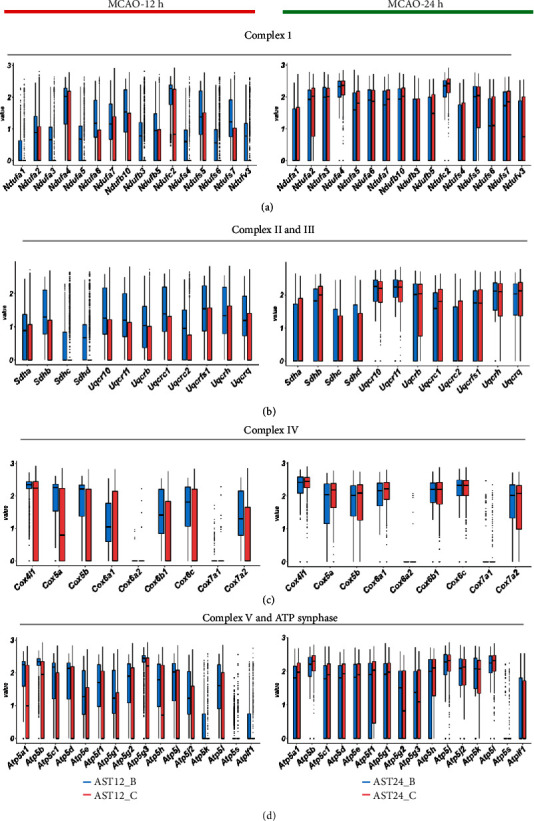
Comparison of the major gene expression in respiratory chain at 12 h and 24 h after MCAO. (a). Boxplots indicated the change of expression for the complex I in mitochondria. (b). Boxplots indicated the change of expression of genes for the complex II and III. (c). Boxplots displayed the change of expression of genes for the complex IV. (d). Boxplots displayed the change of expression of genes for the complex V and ATP syntheses.

**Figure 9:**
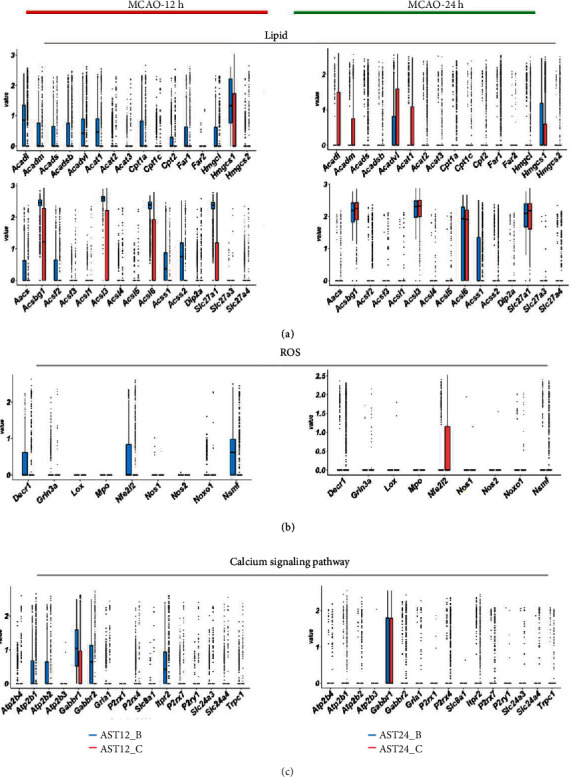
Comparison of the major gene expression in lipid metabolism, reactive oxidative (ROS), and calcium signaling pathways in astrocytes at 12 h and 24 h after MCAO. (a). Boxplots indicated the change of expression of lipid metabolism. (b). Boxplots indicated the change of expression of genes for the ROS. (c). Boxplots displayed the change of expression of genes for calcium signaling pathways.

**Figure 10:**
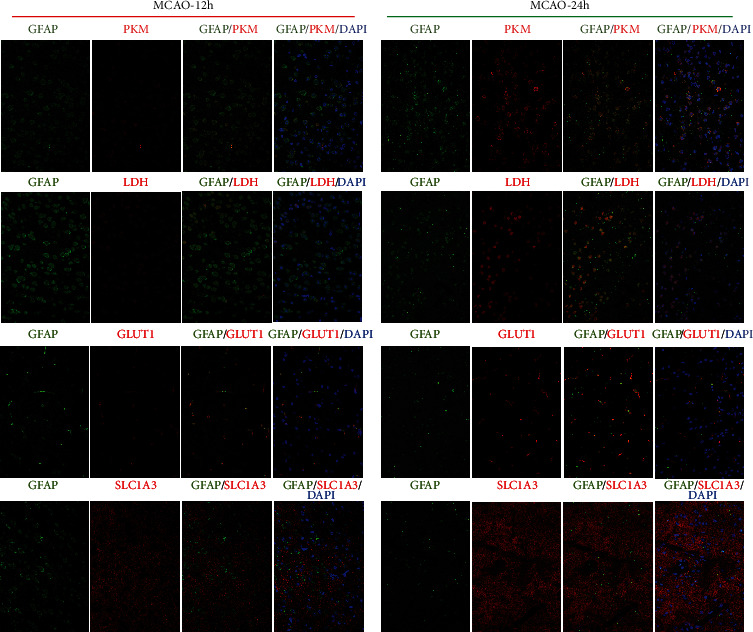
The immunofluorescence images for the 12 h-tMCAO-system and 24 h-tMCAO-system. Validation of differential expression genes related to glycolysis (PKM, upregulation in 24 h), lactate syntheses (LDH, upregulation in 24 h), glucose transporter (GLUT1, upregulation in 24 h), and glutamate transporter (SLC1A3, no significant differences between 12 h and 24 h).

To reveal the dynamic change of astrocytic energy metabolism after ischemic stroke, gene set variation analysis (GSVA) method was performed to evaluate the metabolic pathways regarding glycogen, glucose transportation, glycolysis, pentose phosphate pathway (PPP), lactate metabolism, tricarboxylic acid cycle (TCA), and fatty acid metabolism. In the 12 h-tMCAO-system, the heatmap suggested that all the energetic metabolism pathways downregulated in AST12_C, suggesting that the reactive astrocytes remained energetic dysfunction at 12 h after reperfusion. In contrast, this situation was inversed in the 24 h-tMCAO-system, where astrocytes identified as AST24_C displayed high expression in all energetic metabolism pathways, indicating the reactive astrocytes could have been recovered from the energetic dysfunction state. (Figure 6(a)). The similar changes in the key enzyme of glycolysis, PPP, lactate metabolism and glucose transportation, TCA, respiratory chain and ATP synthase, and fatty acid metabolism were displayed with boxplots (Figures 6(b), 6(c), and 6(d); Figure 7, Figure 8 and Figure 9(a)). Genes for key enzymes of glycolysis tended to be depressed at 12 h and upregulated at 24 h, especially the key genes for pyruvate syntheses such as *Pkm*, and genes for lactate generation such as *Ldha* and *Ldhb*. Double-labeling immunofluorescence verified these changes in the target proteins of *Pkm* and *Ldh* (Figure 10). At 24 h, the key enzymes for fatty acid transportation and consumption greatly increased, indicating the fatty acid metabolism may function as an important energy provider in this stage. We further evaluated the genes expression of glucose and lactate transporters. Among the possible regulatory genes for glucose transportation, *Slc2a1*, which is the regulatory gene for GLUT1, was highly expressed in normal astrocytes, but vastly decreased in 12 h after ischemic stroke, and upregulated at 24 h (Figures 6(d) and 10). This verified the previous assumption of the change in astrocytic energy metabolism state. However, the regulatory genes for lactate transporters were seldom expressed, suggesting the exporter of lactate in astrocytes may not be as active as previously reported. The overproduction of lactate in astrocytes may not increase the lactate concentration in the penumbra after ischemic stroke. In addition, in the early stage, there were no significant differences in the expression of *Glul*, *Slc1a2*, and *Slc1a3*, which suggested that glutamate metabolism was highly activated both at 12 h and 24 h after reperfusion. (Figure 7(d)) The increasing expression of glutamic transporter had also been verified by the double-labeling immunofluorescence. Glutamic could also become a potential energy source at cerebral ischemia.

### 3.6. Dynamic Change of Other Key Roles of Reactive Astrocytes in Ischemic Stroke

**Figure 11:**
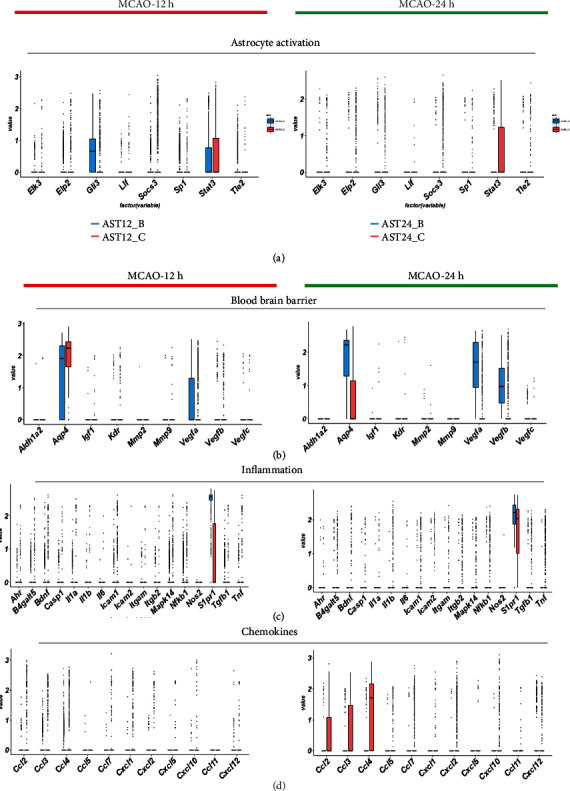
Comparison of the major gene expression in gliosis, blood-brain barrier, and inflammation pathways in astrocytes at 12 h and 24 h after MCAO. (a). Boxplots indicated the change of expression of gliosis. (b). Boxplots indicated the change of expression of genes for the blood-brain barrier. (c). Boxplots displayed the change of expression of genes for inflammation. (d). Boxplots displayed the change of expression of genes for chemokines.

We further analyzed the major genes expression of reactive astrocytes in reactive oxidative stress (ROS) (Figure 9(b)), calcium overload (Figure 9(c)), gliosis (Figure 11(a)), blood-brain barrier (BBB) function (Figure 11(b)), and inflammation (Figure 11(c)). We found that *Aqp4* upregulated at 12 h but vastly decreased at 24 h, possibly indicating self-protection of astrocytes from swelling and edema after ischemia. The decreased expression of *Vegfa* and *Vegfb* probably leads to a decrease in the repair and proliferation of BBB. Next, we analyzed the expression of genes related to inflammation in our database. Although the volcano plot suggested that some genes for chemokines and inflammation were possible signature markers for reactive astrocytes, we did not find obvious upregulation of these genes, except for *S1pr1*. Similarly, we did not find obvious changes of genes for ROS and calcium signaling pathway, which were not in line with our previous assumption. These findings could have been due to the natural low expression of these genes, or these metabolic procedures had not been reaching the highly active states from 12 h to 24 h after ischemic stroke.

## 4. Discussion

We presented a comprehensive characterization of astrocytes in ischemic mice brain cortex after ischemic stroke. Combined with the previously published single-cell sequencing data by Zheng et al. [18], the database with different time spots after ischemic stroke was constructed (12 h and 24 h post MCAO). Our study suggested that there were significant differences in the functional characterization of the reactive astrocytes between 12 h and 24 h. We further revealed the dynamic change of energy metabolism in astrocytes at 12h and 24 h after tMCAO. These results suggested that most energy metabolism was depressed at 12 h and recovered at 24 h. Our work may lay the foundation for developing and applying astrocytic-targeted strategies for neuroprotection treatment for ischemic stroke.

Previous analysis of reactive astrocytes provided important insights into the cell heterogeneity in different pathogenesis [17]. Our study did not support the binary divisions of reactive astrocytes as neurotoxic and neuroprotective, or A1 and A2 types in ischemic stroke. From the perspective of the single-cell scale, we did not identify an evident subpopulation of reactive astrocytes, and we found that their function was dynamic changing in different time spots. We preferred to recognize that the heterogeneity of reactive astrocytes in ischemic stroke was derived from the dynamic transformation of the characterization of the same cell population rather than the differentiation of independent cell subsets. Meanwhile, our study indicated that the characteristic transformation could be sharp in a short period exactly as 12 hours. The astrocytes tend to play an independent role in neuroprotection or adaption in the local ischemic-reperfusion regions at 12 hours, indicated by the activation of pathways including oxidative phosphorylation, gap and tight junction, and ferroptosis. However, the astrocytes at 24 h may prefer to act as signal amplifiers and release inflammation signals such as cytokines to attract assistance from distal places. In addition, the astrocytes at the 12 h and 24 h also share the same activated pathways such as AGE-RAGE and HIF-1 pathway. The interaction and relationship of the two signaling pathways had been reported in the glycolysis process and oxidative stress [20, 21].

Furthermore, our study suggested that the expression of specific-gene for energy metabolism was distinct in astrocytes between 12 h and 24 h. We found that the key enzymes for glycolysis and transporter for glucose were all significantly decreased at 12 h, suggesting a reducing lactate production in astrocytes at this stage. Despite the enzymes of the production of lactate were upregulated at 24h, the expression of genes for the transporter of lactate might not increase equivalently at 24 h, which meant the lactate released to the extracellular matrix was insufficientand, potentially exacerbated the persistent energy deficiency in neurons after reperfusion. Previous research had demonstrated the beneficial effects of lactate administration and suggested the protective role for endogenous lactate production [22–24]. Therefore, the upregulation of lactate transporter in reactive astrocytes is a potential target to reduce ischemic damage. Besides the change of metabolic genes, our study also revealed the possible protective targets at other pathological procedures such as BBB regulation.

In the past decades, there were numerous neuroprotective strategies failing to show benefit in the treatment of acute ischemic stroke [25]. Although thousands of pathological targets were reported, the cognition of accurate intervention for specific cell subtypes at the specific time spot was lacking. Our study had revealed the significant pathway changes of reactive astrocytes at two different time nodes at the acute phase after ischemic stroke. Therefore, more accurate targets could be detected and applied in the neuroprotection strategy.

Our study has limitations. First, this study was based on the comparison of data from the published database and our sequencing results; the heterogeneity between animals and trial conditions could have influenced the analyses results. Second, the findings in our study were based on single-cell RNA-sequencing data, so further verification was manipulated. Third, the astrocytes in the brain were not an isolated cell system, and they had many interactions with other subtypes of brain cells, especially the neurons. Further analysis should be conducted to illustrate the function of astrocytes in the whole brain environment.

## 5. Conclusions

This study provided a new single-cell RNA-sequencing landscape of mice brain at 12 h after ischemic stroke and a novel cognition of astrocyte heterogeneity. The dynamic change of energetic metabolism and major activated pathways in astrocytes at the acute phase of ischemic stroke were presented. This study also revealed the importance of timely intervention of astrocytes and provided potential astrocytic targets for neuroprotection.

## Figures and Tables

**Table 1:** Genes for metabolic procedures.

Metabolic procedures	Genes
Glycolysis	Aldoa, Aldob, Aldoc, Eno1, Eno2, Eno3, Gapdh, Gapdhs, Gck, Gdp1, Gpi1, Hk1, Hk2, Hk3, Mch1, Pfkm, Pfkl, Pfkp, Pgam1, Pgam2, Pgk1, Pkm, Pkm2, Pklr, Tpit
Tricarboxylic acid cycle	Aco2, Cs, Dlst, Dld, Fh1, Idh3a, Idh3b, Idh3g, Idh2, Ogdh, Pcx, Sdha, Sdhb, Sdhc, Sdhd, Suclg1, Suclg2, Suclg3
Intracellular transition of metabolic from glycolysis to tricarboxylic acid cycle	Dlat, Got1, Got2, Mdh1, Mdh2, MPC1, MPC2, Pdha1, Pdha2, Pdhb, Pdhx, Pdk1, Pdk2, Pdk3, Pdk4, Ppm2c
Oxidative phosphorylation	Atp5a1, Atp5b, Atp5c1, Atp5d, Atp5e, Atp5j, Atp5o, Atp5f1, Atp5g2, Atp5g3, Atp5h, Atp5k, Atp5j2, Atp5l, Atp5s, Atpif1, Cox4i1, Cox5a, Cox5b, Cox6a1, Cox6a2, Cox6b1, Cox6c, Cox7a1, Cox7a3, Ndufa1, Ndufa2, Ndufa3, Ndufa4, Ndufa5, Ndufa6, Ndufa7, Ndufb3, Ndufb5, Ndufb10, Ndufc2, Ndufs4, Ndufs5, Ndufs6, Ndufs7, Ndufv3, Sdha, Sdhb, Sdhc, Sdhd, Uqcrb, Uqcrc1, Uqcrc2, Uqcrfs1, Uqcrh, Uqcrq, Uqcr10, Uqcr11
Lactate metabolism	Ldha, Ldhal6b, Ldhb, Ldhc, Slc16a1, Slc16a3, Slc16a4, Slc16a7
Pentose phosphate pathway (PPP)	G6pdx, Pgls, Pdg, Rpe, Rpia, Taldo1, Tkt
Glycogen metabolism	Ugp2, Gyg, Gys1, Gys2, Gsk3a, Gsk3b, Gbe1, Pygl, Pygm, Pygb, Agl
Glucose transport	Slc2a1, Slc2a2, Slc2a3, Slc2a4, Slc2a5, Slc2a6, Slc2a7, Slc2a8, Slc2a9, Slc2a10, Slc2a11, Slc2a12
Glutamic metabolism	Slca1, Slc1a2, Slc1a3, Slc1a6, Slc1a7, Slc38a1, Glul, P2rx7, Grin1, Grin2a, Grin2b, Grin2c, Nsmf
Fatty acids *β*-oxidation	Acadl, Acadm, Dcer1, Dci, Echs1, Gcdh, Hadh, Hadha, Hadhb
Fatty acids synthesis	Acaa2, Acaca, Acacb, Acsl1, Acsl2, Acsl3, Acsl4, Acsl5, Acsl6, Acss2, Acly, Decr1, Ech1, Echdc1, Echdc2, Echdc3, Echs1, Fasn, Mecr, Pecr, Scd1
Triacylglyceride synthesis	Agps, Agpat1, Agpat2, Agpat3, Agpat4, Agpat5, Ayr1, Dgat1, Dgat2, Gk2, Gnpat, Gyk, Gpam, Gpd1, Lpl, Lipe, Lipc, Lipf, Mogat1, Mogat2, Mogat3, Pnpla2, Ppap2a, Ppap2b, Ppap2c

## Data Availability

The data that support the findings of this study are available from the corresponding author upon reasonable request.
